# Genomic Comparisons Revealed the Key Genotypes of *Streptomyces* sp. CB03234-GS26 to Optimize Its Growth and Relevant Production of Tiancimycins

**DOI:** 10.3390/bioengineering11111128

**Published:** 2024-11-08

**Authors:** Huiming Liu, Jing Lin, Yong Huang, Yanwen Duan, Xiangcheng Zhu

**Affiliations:** 1 Xiangya International Academy of Translational Medicine, Central South University, Changsha 410013, China; LHM19930504@csu.edu.cn (H.L.); jonghuang@ihm.ac.cn (Y.H.); 2 National Engineering Research Center of Combinatorial Biosynthesis for Drug Discovery, Changsha 410013, China; 3 Hunan Engineering Research Center of Combinatorial Biosynthesis and Natural Product Drug Discovery, Changsha 410013, China; 4 Muyuan Laboratory, Zhengdong New District, Zhengzhou 450047, China

**Keywords:** *Streptomyces*, tiancimycins, genome resequencing, genotypes, bioinformatics analysis, alkaline tolerance

## Abstract

Strain robustness and titer improvement are major challenges faced in the industrial development of natural products from *Streptomyces*. Tiancimycins (TNMs) produced by *Streptomyces* sp. CB03234 are promising anticancer payloads for antibody-drug conjugates, but further development is severely limited by the low titer of TNMs. Despite many efforts to generate various TNMs overproducers, the mechanisms underlying high TNMs production remain to be explored. Herein, genome resequencing and genomic comparisons of different TNMs overproducers were conducted to explore the unique genotypes in CB03234-GS26. Four target genes were selected for further bioinformatic analyses and genetic validations. The results indicated that the inactivation of histidine ammonia-lyase (HAL) showed the most significant effect by blocking the intracellular degradation of histidine to facilitate relevant enzymatic catalysis and thus improve the production of TNMs. Additionally, the potassium/proton antiporter (P/PA) was crucial for intracellular pH homeostasis, and its deficiency severely impaired the alkaline tolerance of the cells. Subsequent pan-genomic analysis suggested that HAL and P/PA are core enzymes that are highly conserved in *Streptomyces*. Therefore, HAL and P/PA represented novel targets to regulate secondary metabolism and enhance strain robustness and could become potential synthetic biological modules to facilitate development of natural products and strain improvement in *Streptomyces*.

## 1. Introduction

**Figure 1:**
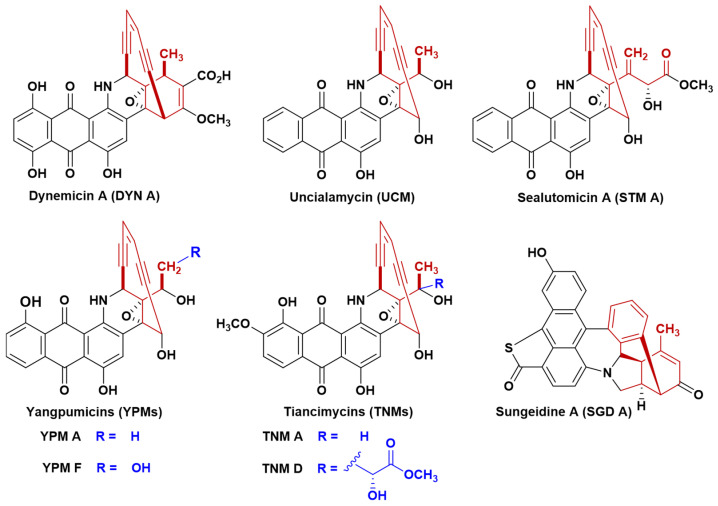
The structures of anthraquinone-fused ten-membered enediynes.

Tiancimycins (TNMs) produced by *Streptomyces* sp. CB03234 [1,2] are anthraquinone-fused ten-membered enediynes (AFEs), and they are categorized altogether with dynemicins (DYNs), uncialamycin (UCM), yangpumicins (YPMs), sealutomicins (STMs), and non-canonical aromatized sungeidines (SGDs) [3] (Figure 1). AFEs represented by TNMs have exhibited high cytotoxicities against various tumors [4,5] and possess a promising potential as payloads for the development of antitumor antibody–drug conjugates (ADCs), but their further medicinal applications are hindered by original low yields. The biosynthetic gene clusters (BGCs) of AFEs share similarities among core genes and regulatory genes, and the corresponding biosynthetic studies have revealed that the general structural scaffold of AFEs could be generated via a unified biosynthetic pathway [6], which might be controlled by a common pathway-specific regulatory mechanism [7]. Due to the notable antitumor activities of TNMs, various approaches have been successively employed to improve the titer of TNMs, including ribosome engineering [8], genome shuffling [9], transcriptional regulation [7,10] and metabolic pathway engineering [11,12]. Therefore, exploring the high-yield mechanisms of TNMs could not only shed new insights to further enhance the production of TNMs, but also provide solid bases for titer improvement of other AFEs and generation of their novel analogs.

*Streptomyces* are important industrial microorganisms producing various secondary metabolites with significant social and economic value [13,14], but how to improve the production of bioactive natural products in *Streptomyces* has been a constant challenge [15]. Metabolic engineering could effectively optimize natural product biosynthesis by reconfiguration of metabolic network, but its feasibility largely depends on the comprehensive understanding of the metabolic and genetic backgrounds of target strains [16,17]. In many cases, without fundamental knowledge, the initial breeding of high-producing strains is still conducted through traditional methods such as random mutagenesis and ribosome engineering [18,19]. Bioinformatics-based inverse metabolic engineering is a conjunctive strategy that coordinately connects traditional strain breeding with metabolic engineering [20,21]. Genome resequencing offers a cost-effective solution to comprehensively analyze genomic data of different mutants based on the reference genome. Genome resequencing in tandem with bioinformatic analyses could reveal the genotype–phenotype relationships of high-producing mutants and then their underlying high-yield mechanisms, so those beneficial genetic targets could be screened out for further rational metabolic engineering and resulting strain or titer improvements [22,23]. For instance, by genomic comparison of the classical rifamycin B overproducer with its reference strain and subsequent filtering of variants, *mutB2*, coding for methylmalonyl-CoA mutase large subunit was successfully validated as the causative target for the overproduction of rifamycin [24]. With the similar strategy, three genes related to primary carbon metabolism were screened and used as the genetic targets for the further enhancement of milbemycin production in *Streptomyces bingchenggensis* [25]. These cases suggest that the genome resequencing-guided inverse metabolic engineering could be a feasible approach to practically improve the production of various microbial natural products and help us to explore the genetic and metabolic regulations governing these productions.

Sequence similarity networks (SSNs) and pan-genomic analysis have recently emerged from big data mining bioinformatic techniques. SSNs construct network models based on similarities between protein or gene sequences [26], revealing relationships and functional differentiation within a gene or protein family [27]. This approach is widely used to identify or predict potential functions of unknown proteins and genes and to reveal evolutionary relationships within protein families [28]. Pan-genomic analysis encompass all genetic information within a certain species, including the core genes that are shared by all strains, the variable genes that are found only in some strains, and the specific genes unique to individual strain [29]. Therefore, pan-genomic analysis could facilitate the annotation and classification of a vast number of genes within the target genome, and the core genes identified by this analysis are particularly valuable for the study of primary metabolism [30]. In our previous study, pan-genomic and transcriptomic studies of *S.* sp. CB03234-S have mined key metabolic targets from numerous genes, and guided a rational reconstitution of fatty acid degradation pathway to facilitate CoA flux enrichment and improve the titers of TNMs [11]. Therefore, the advanced bioinformatic approaches like SSNs and pan-genomic analysis could greatly help us to deepen understanding of targets screened from genome resequencing and guide further metabolic engineering.

*S.* sp. CB03234-GS26 is a TNMs overproducer obtained through genome shuffling of the parental ribosome engineering mutants CB03234-G and CB03234-S [8,9]. Despite of its high TNMs titer, CB03234-GS26 has behaved differently from its parent CB03234-S and exhibited some unfavorable phenotypic features, such as the poor tolerance in nitrogen-rich OP medium and a lengthy fermentation period of over 15 days. Unlike the extensively studied CB03234-S, CB03234-GS26 could possess a distinct high-yield mechanism, which is worth exploring to gain insights for further strain and titer improvements. Herein, the genomic resequencing and comparisons of the above TNMs overproducing mutants shall be conducted to find out unique mutations and corresponding gene targets in CB03234-GS26; and the potential relationship between key genotypes and phenotypes of CB03234-GS26 will be explored through bioinformatic analyses and genetic validations, thereby revealing new targets to fulfill future titer improvements of TNMs and other AFEs, and facilitating development of other natural products in *Streptomyces*. 

## 2. Materials and Methods

### 2.1. Strains and Culture Conditions

All bacterial strains and plasmids used in this study are listed in Table S1, and all designed primers are listed in Table S2. *Streptomyces* sp. CB03234, CB03234-G, CB03234-S (preservation number CCTCC M2017538) and CB03234-GS26 (preservation number CCTCC M2018485) were preserved in our laboratory. These strains were grown on ISP4 solid medium (10 g/L soluble starch, 1 g/L K_2_HPO_4_, 2 g/L (NH_4_)_2_SO_4_•6H_2_O, 1 g/L NaCl, 1 g/L MgSO_4_•7H_2_O, 0.001 g/L FeSO_4_•7H_2_O, 0.001 g/L MnCl_2_•4H_2_O, 2 g/L CaCO_3_, and 20 g/L agar) or G1 solid medium (20 g/L soluble starch, 0.5 g/L K_2_HPO_4_, 1 g/L KNO_3_, 1 g/L NaCl, 1 g/L MgSO_4_•7H_2_O, 0.01 g/L FeSO_4_•7H_2_O, and 20 g/L agar) at 30 °C for sporulation, and grown on mannitol soya flour (MS) solid medium (20 g/L mannitol, 20 g/L soybean flour and 15 g/L agar) at 30 °C for conjugation [9,31]. The Tryptic Soy Broth (TSB) seed medium (17 g/L trytone, 3 g/L soy peptone, 2.5 g/L K_2_HPO_4_, 2.5 g/L glucose and 5 g/L NaCl), OP production medium (15 g/L soluble starch, 15 g/L yeast extract, 2 g/L CaCO_3_, 0.05 g/L CuSO_4_•5H_2_O and 0.005 g/L NaI) and 4FP production medium (60 g/L soluble starch, 20 g/L pharmamedia, 2 g/L CaCO_3_, 0.05 g/L CuSO_4_•5H_2_O and 0.005 g/L NaI) were adopted for TNMs production as previously reported [8,9]. *Escherichia coli* DH5α and S17-1 were used for cloning and conjugation, respectively [31]. *E. coli* strains were grown on Luria–Bertani (LB) medium (10 g/L tryptone, 5 g/L yeast extract and 10 g/L NaCl; for solid medium 15 g/L agar was added) at 37 °C. Antibiotics (50 mg/L apramycin, 25 mg/L nalidixic acid, 50 mg/L kanamycin and 20 mg/L thiostrepton) were added when necessary. All common biological and chemical regents were obtained from standard commercial sources.

### 2.2. Genome Resequencing and Subsequent Bioinformatic Analyses

Genomic DNA extraction was performed using the SteadyPure Bacterial Genomic DNA Extraction Kit (Accurate Biology, Changsha, China). Genome resequencing was conducted by Biomarker Technologies Co., Ltd. (Beijing, China), and the resequencing quality of each mutant was presented in Table S3. The Burrows–Wheeler Aligner was employed to align the resequencing reads with the reference genome of CB03234 (NCBI accession Number: NZ_LIYH00000000) to identify genomic variations in different mutants. The screened mutations were verified through PCR amplification and Sanger sequencing (Tsingke Biotech. Co., Changsha, China) (Figure S1). Based on the Pfam, TIGRFAM, and TMHMM databases, the putative functions of target genes were annotated by Hidden Markov Models using InterProScan v5.35-74.0 [32].

### 2.3. Construction of Different Recombinant Strains

Normal DNA manipulations and molecular biology techniques were performed following standard protocols [33] or commercial procedures. Plasmid extraction, DNA purification, gel extraction, and PCR reactions were performed using commercial kits (Tsingke). Oligonucleotide synthesis and DNA sequencing were also carried out by Tsingke. To construct gene knockout plasmids pOJ260-*ΔHAL^3234^*, pOJ260-*ΔGNAT^3234^* pOJ260-*ΔFNBP^3234^*, and pOJ260-*ΔP/PA^3234^*, the upstream and downstream arms flanking the target gene (approximately 2.0 kb each), as well as the 1490 bp kanamycin-resistant gene (*kan*) or the 919 bp thiostrepton-resistant gene (*tsr*) under the control of the constitutive promoter kasOp* were amplified using the high-fidelity Golden PCR Mix TSE101 (Tsingke). These gene fragments were joined together and cloned into pOJ260 via *Xba*I and *Hin*dIII sites using the Trelief SoSoo seamless cloning kit (Tsingke) to generate the corresponding gene knockout plasmids. The resulting plasmids were validated by DNA sequencing and then introduced into CB03234-S via the *E. coli* S17-1 mediated conjugation transfer as previously described [31]. The kanamycin-resistant (Kan^R^) or thiostrepton-resistant (Tsr^R^) and apramycin-sensitive (Apr^S^) exconjugants were verified through PCR amplification to obtain the desired gene knockout mutants S-*∆HAL^3234^*, S-*∆GNAT^3234^*, S-*∆FNBP^3234^*, and S-*∆P/PA^3234^*. To construct gene overexpression plasmids pSET152-*HAL^3234^*, pSET152-*GNAT^3234^*, pSET152-*FNBP^3234^*, and pSET152-*P/PA^3234^*, each target gene was amplified and cloned into linearized pSET152 via *Xba*I and *Bam*HI sites using the seamless cloning kit. The constructed plasmids were introduced into CB03234-GS26 to generate the expected overexpression mutants GS26-*HAL^3234^*, GS26-*GNAT^3234^*, GS26-*FNBP^3234^*, and GS26-*P/PA^3234^*. 

### 2.4. Fermentation Production and HPLC Analysis of TNMs

In general, 100 μL of spore suspension was inoculated into 50 mL of liquid TSB seed medium and grown at 30 °C and 220 rpm until optimal cell density was achieved (typically 36–48 h). Subsequently, 10% (*v*/*v*) of the seed culture was transferred into 50 mL production medium and incubated under the same conditions for approximately 7 days (for OP medium) or 15 days (for 4FP medium). The fermentation broth was centrifuged at 4000 rpm for 10 min to collect the mycelia and resins, which were then treated as previously described [8]. The collected mycelia and resins were treated ultrasonically three times with a 50% methanol/50% ethyl acetate solution (50 mL, 10 min each time). The combined extracts were then evaporated under vacuum and redissolved in 2 mL methanol. Samples were filtered through a Millipore organic membrane (syringe filter, 0.22 μm) and analyzed on a Waters E2695 HPLC system equipped with a photo-diode array detector and a Welch AQ-C18 column (5 μm, 250 × 4.6 mm, Welch Materials Inc., Shanghai, China). The mobile phase consisted of A (H_2_O) and B (CH_3_CN) at a flow rate of 1 mL/min. A gradient program (90% A for 1 min; 90% A to 5% A for 17 min; 5% A for 2 min; 5% A to 90% A for 3 min, followed by 90% A for 2 min) was applied to detect TNM-A and TNM-D at 540 nm. All fermentation experiments were performed at least in triplicate. 

### 2.5. Sequence Similarity Network Analysis and Streptomyces-Based Pan-Genomic Analysis

Using potassium/proton antiporter (P/PA) (WP_073757435.1) from CB03234 as the query, a BlastP search was conducted on the nonredundant protein sequence database of NCBI (until 5 January 2024) and the sequences of the top 5000 potential P/PA homologues were downloaded. These P/PA sequences were further filtered to remove 216 sequences from unclassified species. The resulting 4784 representative sequences were used to construct the P/PA database using the formatdb command in Blast+. Each P/PA sequence was then queried against the entire database using BlastP with the E-value threshold set at 1.0 × 10^−250^. Duplicates between two nodes and self-loops within each node were removed from the network. The resulting sequence similarity network (SSN) analysis results were visualized using Cytoscape 3.16 [34]. As previously reported [11], a total of 167 complete *Streptomyces* genomes including CB03234 were downloaded from the NCBI Reference Sequence Database (RefSeq). An all-against-all BLASTp analysis of these genomes was performed using blast 2.12.0 with an E-value < e^−10^ for genome-to-genome comparison. The homologues of the target gene presented in the *Streptomyces* genomes were clustered and counted with SILIX [35], with alignment identity and coverage values (IC values) set at ≥50%, 60%, 70%, and 80%, respectively.

## 3. Results and Discussion

### 3.1. Genome Resequencing and Genomic Comparison of CB03234-G, CB03234-S and CB03234-GS26

**Figure 2:**
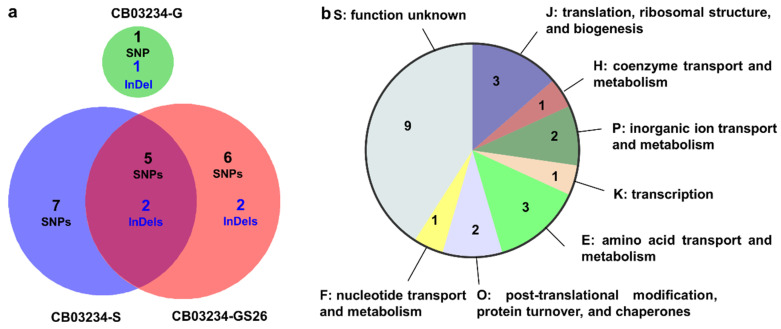
Genome resequencing results of CB03234-G, CB03234-S, and CB03234-GS26. (**a**) The number of mutations in each mutant. (**b**) COG functional classification of 22 mutated CDSs.

The genomes of CB03234-GS26, as well as its parental strains CB03234-G and CB03234-S, were resequenced (Table S3) and aligned with the reference genome of CB03234. As a result, 4, 21, and 26 single nucleotide polymorphism mutations (SNPs), as well as 3, 5 and 6 insertion/deletion mutations (InDels), were detected in CB03234-G, CB03234-S and CB03234-GS26, respectively. These SNPs and InDels were distributed across intergenic regions and coding sequences (CDSs). Since all intergenic mutations were located far away from the promoter regions of CDSs and appeared to have no direct effect, only nonsynonymous SNPs and InDels in CDSs were verified through PCR amplification and Sanger sequencing to confirm their presence in the three mutants (Figure S1). The identified 24 mutations involved a total of 22 CDSs, in which 2 variations (1 SNP and 1 InDel) relating to 2 CDSs were identified in CB03234-G, 14 variations (12 SNPs and 2 InDels) relating to 13 CDSs were identified in CB03234-S, 15 variations (11 SNPs and 4 InDels) relating to 14 CDSs were identified in CB03234-GS26; while 7 variations (5 SNPs and 2 InDels) relating to 7 CDSs were shared by CB03234-S and CB03234-GS26 (Figure 2a, Table 1). These 22 mutated CDSs were classified according to the Clusters of Orthologous Groups (COGs) of protein database in NCBI (https://www.ncbi.nlm.nih.gov/research/cog/, accessed on 20 September 2022). Among them, nine CDSs belonged to the function unknown category, seven CDSs belonged to various transport and metabolism categories, one CDS belonged to the transcription category, two CDSs belonged to the post-translational modification category and three CDSs belonged to the translation category (Figure 2b). The comparison of genomic variations from three mutants suggested that CB03234-GS26 was more correlated to CB03234-S.

### 3.2. Bioinformatic Analyses and Functional Prediction of the Mutated Target Genes in CB03234-GS26

Besides the seven genomic variations shared with CB03234-S, CB03234-GS26 also possessed eight unique mutations involving seven genes. As shown in Table 1, both AMK26_RS09465 and AMK26_RS17285 encode hypothetic proteins, AMK26_RS31405 encodes a SAM-dependent methyltransferase but locates in the biosynthetic gene cluster of type-III polyketide flaviolin, which is naturally silenced in CB03234 and related mutants. Therefore, the mutations on these three genes seem to have no direct effect on central metabolisms and production of TNMs. On the other hand, AMK26_RS08900 encodes a histidine ammonia-lyase (HAL^3234^), AMK26_RS16830 encodes a GNAT family N-acetyltransferase (GNAT^3234^), AMK26_RS27145 encodes a FAD/NAD (P)-binding protein (FNBP^3234^), and AMK26_RS27375 encodes a potassium/proton antiporter (P/PA^3234^). These genes may be involved in metabolic or physiological functions and were thus selected for further investigations. The possible impacts of gene mutations on the corresponding proteins were evaluated using the Conserved Domain Search (CD-Search) tool from NCBI (Figure S2). The InDel in GNAT^3234^ was not in the conserved domain region, but it resulted in the loss of nine amino acids (53rd–61th), which might disrupt the three-dimensional structure of GNAT^3234^ to affect its stability and activity. The SNP in *HAL^3234^* caused an A46S substitution in the conserved domain region nearing active sites, potentially affecting the functionality of HAL^3234^. The SNP in *FNBP^3234^* was not located in the core NAD-binding domain and its possible influence was not certain. The two SNPs in *P/PA^3234^* led to V94F and G99D substitutions, which are very close to each other within the conserved domain region and could potentially affect the functionality of P/PA^3234^. 

**Figure 3:**
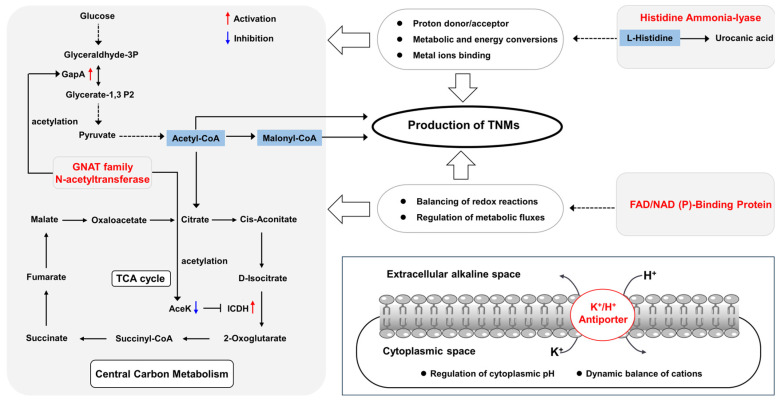
Proposed metabolic or physiological functions of the screened target proteins in CB03234-GS26.

The possible physiological and metabolic roles of the above target proteins were proposed based on the reported researches of their known homologues (Figure 3). In general, GNATs are prevalent in bacteria and mainly catalyze the acetylation of various metabolic enzymes [36,37]. In *Salmonella enterica*, GNATs promote the central carbon metabolism by acetylating glyceraldehyde phosphate dehydrogenase (GapA) and isocitric dehydrogenase (ICDH) kinase/phosphatase (AceK), in which the increased activity of acetylated GapA strengthens the glycolysis pathway, while the decreased activity of acetylated AceK reduces the phosphorylation of ICDH, resulting in the increased activity of ICDH to enhance the TCA cycle [38]. Thus, GNAT^3234^ could propel the central carbon metabolism to compete with the production of TNMs for the CoA fluxes. Histidine could bind to metal ions or act as a proton donor/acceptor to participate various enzymatic reactions including metabolic and energy conversions [39,40], but HALs catalyze the first step of histidine degradation by removing its amino group to form urocanate [41] and thus diminish the intracellular level of histidine. Meanwhile, FNBPs are usually involved with balancing of redox reactions and regulation of metabolic fluxes [42,43]. Therefore, HAL^3234^ and FNBP^3234^ might contribute pleiotropic effects on both primary and secondary metabolisms to indirectly regulation the biosynthesis of TNMs. On the other hand, P/PAs widely exist in various microorganisms and plants, and they usually regulate the mutual exchange of potassium ions and protons to maintain intracellular pH and ions [44,45], so P/PA^3234^ probably serves a similar function to balance pH and ion levels. Based on the above summaries, we proposed that the mutations in the four target genes were more likely to cause physiological and metabolic changes of CB03234-GS26, thereby affecting the production of TNMs.

### 3.3. Genetic Validations of Four Target Genes in CB03234-S and CB03234-GS26

**Figure 4:**
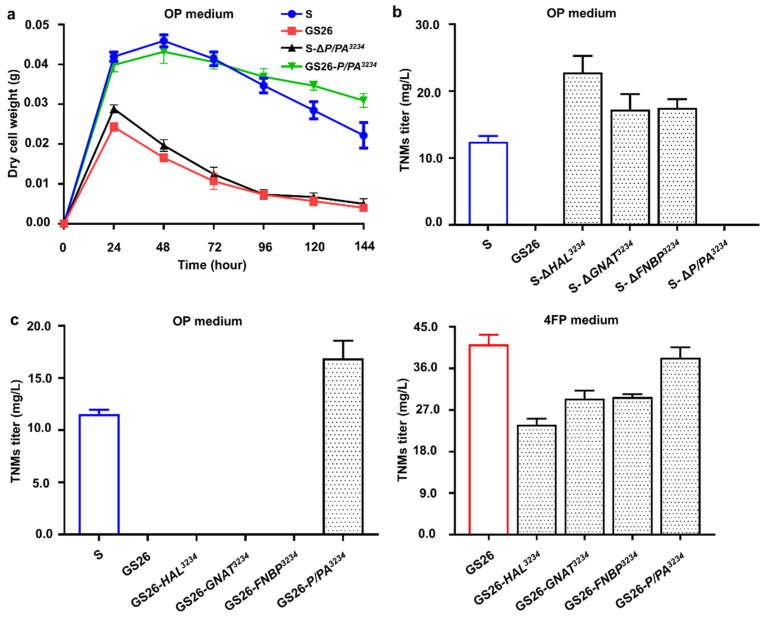
Fermentation characterization of various strains and mutants. (**a**) Growth curves of CB03234-S, CB03234-GS26, and their related mutants cultured in OP medium. (**b**) The TNMs titers of the CB03234-S related gene knockout mutants in OP medium. (**c**) The TNMs titers of the CB03234-GS26 related overexpression mutants in OP and 4FP media, respectively.

First, four target genes were individually inactivated in CB03234-S via homologous recombination (Figure S3). When the acquired mutants were fermented in OP medium, a significant biomass decrease of CB03234-S-*∆P/PA^3234^* was observed, whereas the other mutants showed no different growth states to that of CB03234-S (Figure 4a). Compared to the original 12.8 ± 0.2 mg/L TNMs in CB03234-S, the production of TNMs was almost silenced in CB03234-S-*∆P/PA^3234^* due to its notable biomass reduction, while the titers of TNMs in other three mutants all showed varying degrees of enhancement, and the highest titer of 22.8 ± 2.5 mg/L TNMs was obtained in CB03234-S-*∆HAL^3234^* (Figure 4b). Meanwhile, these target genes were also overexpressed, respectively, in CB03234-GS26 for possible functional complementation. When the acquired mutants were fermented in 4FP medium, their growth states were similar to that of CB03234-GS26, but the productions of TNMs in these mutants were decreased in different degrees (Figure 4c). In contrast to the 41.2 ± 2.1 mg/L TNMs in CB03234-GS26, the TNMs titers in CB03234-GS26-*HAL^3234^*, CB03234-GS26-*GNAT^3234^*, CB03234-GS26-*FNBP^3234^* and CB03234-GS26-*P/PA^3234^* were 23.8 ± 1.3 mg/L, 29.5 ± 1.7 mg/L, 29.8 ± 0.6 mg/L and 38.3 ± 2.3 mg/L, respectively (Figure 4c). Interestingly, when these mutants were fermented in OP medium, only CB03234-GS26-*P/PA^3234^* restored the cell growth (Figure 4a) and enabled the production of TNMs from undetectable to 16.9 ± 1.7 mg/L within 7 days, while other three mutants all behaved like CB03234-GS26 with poor growth and no production of TNMs (Figure 4c).

The above genetic experiments suggested that HAL^3234^, GNAT^3234^ and FNBP^3234^ exerted different levels of negative effects on the production of TNMs, in which HAL^3234^ showed the most significant impact; by contrast, P/PA^3234^ played a crucial role to maintain the alkaline tolerance of cells in nitrogen-rich liquid fermentation but appeared not to be directly related to the production of TNMs. The inactivation of HAL^3234^ blocked the intracellular degradation of histidine, so the resulting accumulation of histidine might beneficial for various enzymatic catalysis in secondary metabolisms and thereby promoted the production of TNMs. We proposed that both GNAT^3234^ and FNBP^3234^ could regulate various metabolic fluxes to assist the central carbon metabolism, so their inactivation could interfere with the primary carbon metabolic network, guiding the acetyl-CoA flux toward secondary metabolism to strengthen TNMs biosynthesis. On the other hand, P/PA^3234^ could expel internal potassium ions in exchange for external protons, thereby regulating intracellular pH and ion balance to maintain cellular alkaline tolerance. The inactivation of P/PA^3234^ restricts the entry of protons under alkaline environments, while unprotonated amines passively move into cells and induce cytoplasmic alkalinization [46], which in turn risks disrupting diverse cellular processes such as differentiation, proliferation, migration, etc. [47] and causing cell death. Thus, we concluded that the defective P/PA^3234^ makes CB03234-GS26 very vulnerable confronting the alkaline stresses of OP medium, and this case represented the first example validating the physiological function of P/PA in *Streptomyces*. 

### 3.4. Sequence Similarity Network Analysis of P/PA Homologues and Streptomyces-Based Pan-Genomic Analyses of Target Genes

**Figure 5:**
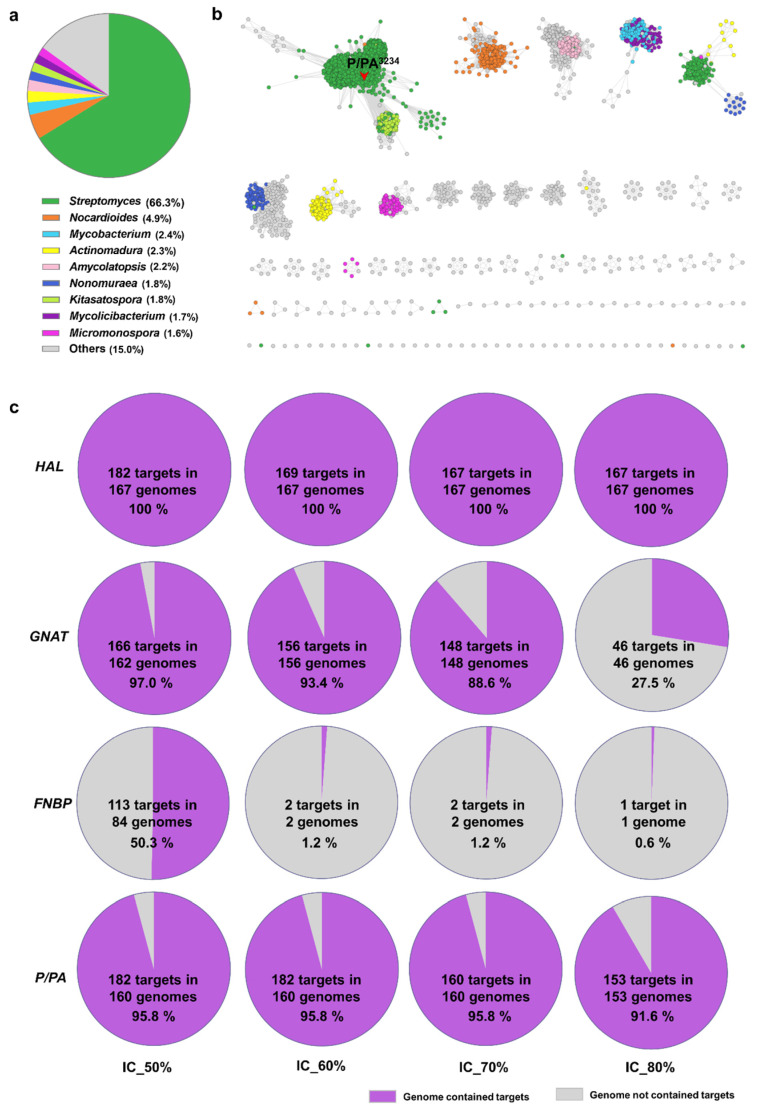
SSN analysis of P/PA homologues and *Streptomyces*-based pan-genomic analyses of target genes. (**a**) the taxonomic distribution and (**b**) the SSN analysis (E value = 1 × 10^−250^) of 4784 P/PA homologues. (**c**) Pan-genomic analyses of four target genes under different IC values.

Due to the important physiological function of P/PA, the corresponding SSN analysis was further carried out to investigate the prevalence of P/PA in *Streptomyces*. Using the P/PA^3234^ sequence as the query, potential P/PA homologues were searched in the nonredundant protein sequence database of NCBI through BlastP. The top 5000 sequences with the criteria of over 85% coverage and 50% identity were downloaded and filtered by classifications to remove the data from unknown species. The screened 4784 P/PA homologues were all originated from *Actinomycetes*, of which 3171 homologues specifically belonged to *Streptomyces*, accounting for about 66.3% of the total (Figure 5a). The SSN analysis results revealed that most P/PA homologues clustered together with a clear species specificity, and over 94% of candidates from *Streptomyces*, including P/PA^3234^, gathered to form the largest cluster, indicating that P/PA homologues were widely distributed across *Streptomyces* and highly conserved (Figure 5b). 

Subsequent pan-genomic analyses of the four target genes in 167 *Streptomyces* genomes were conducted at different identity and coverage values (IC values) (Figure 5c). As a result, *HAL* was present in all *Streptomyces* genomes under every threshold, while *P/PA* was found in over 95% of *Streptomyces* genomes when the IC values ranged from 50% to 70%, and in over 90% of *Streptomyces* genomes even at the IC value of 80%. Therefore, both *HAL* and *P/PA* are core genes highly conserved across *Streptomyces*, and this outcome was consistent with the functional roles of their encoded proteins, where HAL is involved in primary histidine metabolism and P/PA is essential for growth and survival of hosts under alkaline conditions. On the other hand, the presence of *GNAT* in *Streptomyces* genomes gradually decreased with increasing the IC value from 50% to 70%, and dramatically dropped to less than 30% at an IC value of 80%, which suggested that *GNAT* is a variable gene with moderate conservation and similarity in *Streptomyces*. In comparison, the presence of *FNBP* in *Streptomyces* genomes was very low when the IC value was beyond 50%, which meant *FNBP* is a specific gene solely found in CB03234. In summary, our data suggested that HAL and P/PA could become potential synthetic biological modules to facilitate natural product developments and strain improvements in *Streptomyces*, in which the former could be a new target to regulate enzymes involved in microbial secondary metabolism to enhance or activate natural product productions, while the latter could be a new target to improve the alkaline endurance of *Streptomyces* species, especially those industrial producers using nitrogen-rich medium.

## 4. Conclusions

In the present study, genome resequencing of different TNMs overproducing mutants was conducted to explore the genetic bases for the characteristic phenotypes and high-yield mechanism of CB03234-GS26. After comprehensive genomic comparisons and further Sanger sequencing validations, eight unique mutations involving seven genes found in CB03234-GS26 were screened out. Based on the bioinformatics predictions, four putative functional genes were selected for further genetic validations in both CB03234-S and CB03234-GS26. The results suggested that the mutations of HAL^3234^, GNAT^3234^, and FNBP^3234^ exerted differential effects to improve the production of TNMs. Among them, the inactivation of HAL^3234^ showed the most significant impact by blocking the intracellular degradation of histidine to facilitate relevant enzyme catalysis; thus, the TNMs titer was increased for a notable 1.8-fold from original 12.8 ± 0.2 mg/L in CB03234-S to 22.8 ± 2.5 mg/L in CB03234-S-*∆HAL^3234^*. In addition, the defection of P/PA^3234^ was related to the phenotype of CB03234-GS26 by unbalancing the intracellular pH homeostasis to severely impair the alkaline tolerance of the cells, so the growth and TNMs production were restored in CB03234-GS26-*P/PA^3234^* with the shortened fermentation period. Subsequent SSN analysis revealed the prevalence of P/PA across *Streptomyces*, and pan-genomic analysis suggested that HAL and P/PA were highly conserved core enzymes in *Streptomyces*, while GNAT was a variable enzyme in *Streptomyces* and FNBP was a specific enzyme in CB03234. Therefore, HAL and P/PA, respectively, represented a novel target to regulate microbial secondary metabolism and enhance host robustness, and they could become potential synthetic biological modules to facilitate development of natural products and strain improvement in *Streptomyces*.

## Figures and Tables

**Table 1:** CDS variations in CB03234-G, CB03234-S, and CB03234-GS26.

Strains	Locus_Tag	Protein Length (AA)	Mutation	Putative Functions
CB03234-G	AMK26_RS07435	288	295T > TG (frameshift)	zinc metalloprotease HtpX
	AMK26_RS07635	398	329C > A(A110D)	elongation factor Tu
CB03234-S	AMK26_RS10695	396	362C > A(S121T)	superoxide dismutase, Ni
	AMK26_RS14490	446	503C > G(A168G)	uracil permease (purine permease)
	AMK26_RS14790	515	782G > T(S261I) 808G > A(V270I)	D-alanine glycine permease (alanine:cation symporter family protein)
	AMK26_RS26890	259	280C > A(L94M)	transcriptional regulator (Scr1 family TA system antitoxin-like transcriptional regulator)
	AMK26_RS34215	1081	103T > A(T35S)	hypothetical protein
	AMK26_RS07620	124	129C > G(K43N)	30S ribosomal protein S12
CB03234-S/ GS26	AMK26_RS22320	186	266G > C(G89A)	hypothetical protein
	AMK26_RS34270	74	175G > C(R59G)	hypothetical protein
	AMK26_RS10740	518	353G > C(P118R)	alkaline phosphatase D family protein
	AMK26_RS16920	393	513C > A(Y171 *)	agmatine deiminase
	AMK26_RS17525	338	97G > T(K33Q)	hemolysin family protein
	AMK26_RS12260	194	582G > GC (frameshift)	ATP/GTP-binding protein
	AMK26_RS31245	63	(141–143)GGC > G (frameshift)	hypothetical protein
CB03234-GS26	AMK26_RS08900	513	133C > A(A45S)	histidine ammonia-lyase
	AMK26_RS16830	262	(157–184) ACGGGCC GTCCGGGCCGTCC GGGCCGTC > A (codon_deletion)	GNAT family N-acetyltransferase
	AMK26_RS27145	641	1075C > T (G359S)	FAD/NAD (P)-binding protein
	AMK26_RS27375	487	235C > A (V79F) 251C > T (G84D)	potassium/proton antiporter
	AMK26_RS09465	772	(762–295) GCTCCCC GTCGGCGACGAG CGGCCCGTCGACC CG > G (codon_deletion)	hypothetical protein
	AMK26_RS17285	132	339C > A(Y113 *)	hypothetical protein
	AMK26_RS31405	270	161A > T(V54E)	SAM-dependent methyltransferase

## Data Availability

The original contributions presented in the study are included in the article; further inquiries can be directed to the corresponding authors.

## Supplementary Materials

The following supporting information can be downloaded at: https://www.mdpi.com/article/10.3390/bioengineering11111128/s1, Figure S1: Sanger sequencing results of mutations found in CB03234-G, CB03234-S and CB03234-GS26; Figure S2: CD analyses of four target proteins from CB03234-GS26 with the locations of their mutation sites; Figure S3: Construction and PCR validation of gene knockout mutants derived from CB03234-S. **a** Knockout of *HAL^3234^*. **b** Knockout of *GNAT^3234^*. **c** Knockout of *FNBP^3234^*. **d** Knockout of *P/PA^3234^*; Table S1: Strains and plasmids used in this study; Table S2: Primers used in this study; Table S3: The quality of CB03234-G, CB03234-S and CB03234-GS26 genome sequence reads. References [48,49] are cited in the supplementary materials.
