# Supplementary material for: Genomic Comparisons Revealed the Key Genotypes of Streptomyces sp. CB03234-GS26 to Optimize Its Growth and Relevant Production of Tiancimycins
Source: Bioengineering (Basel). 2024 Nov 8;11(11):1128. doi: 10.3390/bioengineering11111128 (PMC11591506; doi:10.3390/bioengineering11111128)
Supplement: Supplementary file 1 [file bioengineering-11-01128-s001.zip › bioengineering-3285459-supplementary.pdf]

Supplementary Information

Genomic Comparisons Revealed the Key Genotypes of *Streptomyces* sp. CB03234-GS26 to Optimize Its Growth and Relevant Production of Tiancimycins

Huiming Liu [1], Jing Lin [1], Yong Huang [1,2], Yanwen Duan [1,2,3,*] and Xiangcheng Zhu [1,2,4,*]

1 Xiangya International Academy of Translational Medicine, Central South University, Changsha 410013, China; LHM19930504@csu.edu.cn (H.L.); jonghuang@ihm.ac.cn (Y.H.)

2 National Engineering Research Center of Combinatorial Biosynthesis for Drug Discovery, Changsha 410013, China

3 Hunan Engineering Research Center of Combinatorial Biosynthesis and Natural Product Drug Discovery, Changsha 410013, China

4 Muyuan Laboratory, Zhengdong New District, Zhengzhou 450047, China

* Correspondence: ywduan66@sina.com (Y.D.); seanzhu1996@aliyun.com (X.Z.); Tel.: +86-731-8265-0539 (X.Z.); Fax: +86-731-8265-0551 (X.Z.)

Table S1. Strains and plasmids used in this study.

Strains/Plasmids	Descriptions	Reference/Source
E.coli strains		
DH5α	General cloning	Commercial source
S17-1	Intergenic conjugal transfer	[1]
Streptomyces strains		
CB03234	Producing strain of tiancimycins	[2]
CB03234-S (CCTCC M2017538)	A Str-directed ribosome engineering mutant of CB03234	[3]
CB03234-GS26 (CCTCC M2018485)	A genome shuffling recombinant from CB03234-G and CB03234-S	[4]
CB03234-G	A Gen-directed ribosome engineering mutant of CB03234	this study
S-Δ*HAL*^{3234}	A derivative strain of CB03234-S with deleted *histidine ammonia-lyase*	this study
S-Δ*GNAT*^{3234}	A derivative strain of CB03234-S with deleted *GNAT family N-acetyltransferase*	this study
S-Δ*FNBP*^{3234}	A derivative strain of CB03234-S with deleted *FAD/NAD (P)-binding protein*	this study
S-Δ*P/PA*^{3234}	A derivative strain of CB03234-S with deleted *potassium/proton antiporter*	this study
GS26-*HAL*^{3234}	CB03234-GS26 integrated with pSET 152-*histidine ammonia-lyase*	this study
GS26-*GNAT*^{3234}	CB03234-GS26 integrated with pSET 152-*GNAT family N-acetyltransferase*	this study
GS26-*FNBP*^{3234}	CB03234-GS26 integrated with pSET 152-*FAD/NAD (P)-binding protein*	this study
GS26-*P/PA*^{3234}	CB03234-GS26 integrated with pSET 152-*potassium/proton antiporter*	this study
Plasmids		
pOJ260	Conjugal shuttle vector	[5]
pOJ260-Δ*HAL*^{3234}	pOJ260 harboring the up and downstream 2.0 kb regions of *histidine ammonis-lyase* integrated with tsr	this study

Strains/Plasmids	Descriptions	Reference/Source
pOJ260-Δ*GNAT*^{3234}	pOJ260 harboring the up and downstream 2.0 kb regions of *GNAT family N-acetyltransferase* integrated with tsr	this study
pOJ260-Δ*FNBP*^{3234}	pOJ260 harboring the up and downstream 2.0 kb regions of *FAD/NAD (P)-binding protein* integrated with kan	this study
pOJ260-Δ*P/PA*^{3234}	pOJ260 harboring the up and downstream 2.0 kb regions of *potassium/proton antiporter* integrated with kan	this study
pSET152	Conjugal shuttle vector	[5]
pSET152-*HAL*^{3234}	pSET152 harboring CB03234-*histidine ammonis-lyase*	this study
pSET152-*GNAT*^{3234}	pSET152 harboring CB03234-*GNAT family N-acetyltransferase*	this study
pSET152-*FNBP*^{3234}	pSET152 harboring CB03234-*FAD/NAD (P)-binding protein*	this study
pSET152-*P/PA*^{3234}	pSET152 harboring CB03234-*potassium/proton antiporter*	this study

Table S2. Primers used in this study.

Primers	Nucleotide sequences 5'-3'
ΔHAL^{3234}-up-for	ACGACGGCCAGTGCCAAGCTTTGTTGCTGATCCCCAGCTTCT
ΔHAL^{3234}-up-rev	GTTCGAATGTGAACAATCCATATTCAGCAGCGTACGG
ΔHAL^{3234}-*tsr*-for	CTGCTGAATATGGATTGTTCACATTCGAACGGTCTCTG
ΔHAL^{3234}-*tsr*-rev	TCCTCCTGCATGTTATCGGTTGGCCGCGAGATT
ΔHAL^{3234}-dn-for	GCCAACCGATAACATGCAGGAGGACCACGTC
ΔHAL^{3234}-dn-rev	GCGGCCGCGGATCCTCTAGAGCTTCAGCGTGACCTTGGAG
ΔHAL^{3234}-check-for	CTCCGGCTATGGGCATCTG
ΔHAL^{3234}-check-rev	CTTCCCCTCCCGTACGAAG
$\Delta GNAT^{3234}$-up-for	ACGACGGCCAGTGCCAAGCTTCAGAAAGATCCCTCCGTGAACC
$\Delta GNAT^{3234}$-up-rev	CGAATGTGAACAGGTTCGGGTGGTGTGG
$\Delta GNAT^{3234}$-*tsr*-for	ACCACCCGAACCTGTTCACATTCGAACGGTCTCTGC
$\Delta GNAT^{3234}$-*tsr*-rev	GCAGCAGGGTGTTTATCGGTTGGCCGCGAGATT
$\Delta GNAT^{3234}$-dn-for	GCCAACCGATAAACACCCTGCTGCTTTACATACAC
$\Delta GNAT^{3234}$-dn-rev	GCGGCCGCGGATCCTCTAGAGGCACAGCCGCTGGATAC
$\Delta GNAT^{3234}$-check-for	CATCAACAACGTCCTCGGCC
$\Delta GNAT^{3234}$-check-rev	GCGAATTCGCCGAGCATCTT
$\Delta FNBP^{3234}$-up-for	CACATTCCACAGAGTCCTCGCAGAGCGACC
$\Delta FNBP^{3234}$-up-rev	GCGGCCGCGGATCCTCTAGACGTCCGGGTCGTCGTTGA
$\Delta FNBP^{3234}$-*kan*-for	TCGACGATCCGGTCAGAAGAACTCGTCAAGAAGGCGA
$\Delta FNBP^{3234}$-*kan*-rev	TCTGCGAGGACTCTGTGGAATGTGTGTCAGTTAGGGTG
$\Delta FNBP^{3234}$-dn-for	ACGACGGCCAGTGCCAAGCTTAGGTGGTGACGGGTCTCG
$\Delta FNBP^{3234}$-dn-rev	GAGTTCTTCTGACCGGATCGTCGATCACTCAGG
$\Delta FNBP^{3234}$-check-for	TGAGGGTGGTCACGGTGACC
$\Delta FNBP^{3234}$-check-rev	GCGCTCGAACTGACCGAAC
$\Delta P/PA^{3234}$-up-for	CACATTCCACAGCCTTCCACTTGGTTCCCAGGC
$\Delta P/PA^{3234}$-up-rev	GCGGCCGCGGATCCTCTAGATTGAAGACGGCGTCCATGGAC
$\Delta P/PA^{3234}$-*kan*-for	CAGGACCACCGATCAGAAGAACTCGTCAAGAAGGCGA
$\Delta P/PA^{3234}$-*kan*-rev	CCAAGTGGAAGGCTGTGGAATGTGTGTCAGTTAGGGT
$\Delta P/PA^{3234}$-dn-for	ACGACGGCCAGTGCCAAGCTTCAGCATCACCACGGAGTGC
$\Delta P/PA^{3234}$-dn-rev	GAGTTCTTCTGATCGGTGGTCCTGGGCAAC

Primers	Nucleotide sequences 5'-3'
Δ*P/PA*^{3234}-check-for	ATGACATCAGGGCCTGCTCC
Δ*P/PA*^{3234}-check-rev	CATCTGCTCGCTCGTGCTG
HAL^{3234}-for	GGCTGCAGGTCGACGCTCTAGAGCCTTCCTCGCGATGGGGACA
HAL^{3234}-rev	TCGTGCCGGTTGGTACGGGATCCCGATGCACACAGTCGTGGTGG
GNAT^{3234}-for	GGCTGCAGGTCGACGCTCTAGAGCTCACAGCAGGTGTCGCAAGG
GNAT^{3234}-rev	ATCGTGCCGGTTGGTACGGGATCCCGGTGCCTCCCACCGATGC
FNBP^{3234}-for	GGCTGCAGGTCGACGCTCTAGAGCTCACGAGGCGTGCGCC
FNBP^{3234}-rev	TCGTGCCGGTTGGTACGGGATCCCGTTGGACGGTCGGCTGAGTGTC
P/PA^{3234}-for	GGCTGCAGGTCGACGCTCTAGAGCCTACCGTCCCCTCCCGTCC
P/PA^{3234}-rev	TCGTGCCGGTTGGTACGGGATCCCGCTGACTGTCCACCAGCTCAACGAA
AMK26_RS07635-for	CCGGCTTGATGATGCACTGG
AMK26_RS07635-rev	GTGGCGAAGGCGAAGTTCGA
AMK26_RS07435-for	TGAGCTTGAAGTTCGCGATGC
AMK26_RS07435-rev	GCGTACTGGAACAGCGACAAG
AMK26_RS10695-for	CTTCACGATGAGCAGGTCCT
AMK26_RS10695-rev	TCTCCCAAACGTCTCCCAAA
AMK26_RS14490-for	AGAAGGCCCGTGTTCCAC
AMK26_RS14490-rev	ATCACGATGCACAGGGAGAG
AMK26_RS14790-for	CGTGGTGAGAGGGAAGTACG
AMK26_RS14790-rev	CGACTTGAGGCCGTAGTAGC
AMK26_RS26890-for	GGATGGCGTCGAAGTAGATT
AMK26_RS26890-rev	ACCTCTTCGGGTTTCGAGAT
AMK26_RS34215-for	GGGGCGTTCATATCCATTGG
AMK26_RS34215-rev	CAGTTCCTCGATCCGGGAC
AMK26_RS07620-for	AGGTTAGCTTTACTACACGGCACA
AMK26_RS07620-rev	TCTTACTTCTCCTTCTTGGCGCC
AMK26_RS22320-for	ACCGTCAGCCGTACGTGTC
AMK26_RS22320-rev	ACCGTCAGCCGTACGTGTC
AMK26_RS34270-for	CGAATGTACAGGCGTCACAC
AMK26_RS34270-rev	GAGGATGTAACTGCCCTTGC
AMK26_RS10740-for	GTAGGAGCGCAGGTCGAG
AMK26_RS10740-rev	CTCCTCGTTTTCACCCGTACC
AMK26_RS16920-for	CAGCTGAGCAGAAGGGTCTT

Primers	Nucleotide sequences 5'-3'
AMK26_RS16920-rev	**GCCGGTCTCGTAGTAGTTGG**
AMK26_RS17525-for	**GAAGGCGTTGATCGTGAAGA**
AMK26_RS17525-rev	**GTACCGACCAGTTGGAGACG**
AMK26_RS12260-for	**GAACTGCTCCCGCTCGAC**
AMK26_RS12260-rev	**GGCTCCCTGTCGACGTAACC**
AMK26_RS31245-for	**ATCGACCTGGAAGTCGTCGT**
AMK26_RS31245-rev	**AGCTGCTTGACGGTGTCC**
AMK26_RS08900-for	**GTCGGTGCCGTTGAGGAG**
AMK26_RS08900-rev	**ACGGTTGGGAGCCGTTAC**
AMK26_RS23160-for	**GGTGTCGAGGGTGTACGTG**
AMK26_RS23160-rev	**GGACACGCGGTAGTGGTTG**
AMK26_RS27145-for	**ATGTCCGAGGACTGGATACG**
AMK26_RS27145-rev	**CCTCGACACGGAAAGATGGAG**
AMK26_RS27375-for	**GAAGTCGTCGATCAGCTCGT**
AMK26_RS27375-rev	**AGTGCTGGAGCAACCATAGG**
AMK26_RS31405-for	**ATGAAGTCGGTCACGAAGGT**
AMK26_RS31405-rev	**ACCACCAGTGGGAACAGC**
AMK26_RS17285-for	**CATCGACTGGCGCATCCC**
AMK26_RS17285-rev	**GGGCTACTGGCCCTTCGT**
AMK26_RS16830-for	**ATGACACGTGTGCAGCTGAG**
AMK26_RS16830-rev	**CGCCTACTACCTCGGCATCAA**
AMK26_RS09465-for	**GGGTGGCTCTTCCGGACAC**
AMK26_RS09465-rev	**CAGGTTCTCCGGCAGCATG**
AMK26_RS00790-for	**CGATCTCCAAACTGAACGCCCTAG**
AMK26_RS00790-rev	**GATCCTGCGCTTCTTCTTCGGGA**
AMK26_RS04500-for	**GGGCTGGTTCTGTACGAGCT**
AMK26_RS04500-rev	**AGTTCACCCTGGAGGGGC**
AMK26_RS07865-for	**CGAAGAGTCGTCCCACAGCT**
AMK26_RS07865-rev	**CCCCGAGAAGGCTGTTGAGA**
AMK26_RS17875-for	**CAGGTCCTCGGGCTGTTC**
AMK26_RS17875-rev	**GCAGCTGGAGCTGGTAGAG**
AMK26_RS05675-for	**CAGGAACCGGTCCACTCC**
AMK26_RS05675-rev	**GGGGGTGAAGTCCATCCTG**
AMK26_RS24170-for	**GTCGAGCACTTCTCCTGGAAG**
AMK26_RS24170-rev	**TAGCACGGGTCGTGGTAAGTG**
AMK26_RS24795-for	**CCTCGATGAGTGAAGTGACGCT**
AMK26_RS24795-rev	**ACACGAGCGAGGAGGGTTAC**
AMK26_RS07435-for	**TGAGCTTGAAGTTCGCGATGC**
AMK26_RS07435-rev	**GCGTACTGGAACAGCGACAAG**
AMK26_RS10870-for	**GACTGTGTCCCCCGTCTCTTC**
AMK26_RS10870-rev	**CGAGGCCAACTCCAACACC**
AMK26_RS11340-for	**GGCAGGTCATGTCGGAACC**

Primers	Nucleotide sequences 5'-3'
AMK26_RS11340-rev	**CGGTGAAGGTGAGCAGGAAGA**
AMK26_RS11455-for	**ACCACGAACAGCATGTACCA**
AMK26_RS11455-rev	**CCAGATGAAACCGTGGGAGAC**
AMK26_RS11565-for	**GTGGAGCTGGGAGTCATGGAG**
AMK26_RS11565-rev	**CAGGTCGTGCTCGAACATCATC**
AMK26_RS11660-for	**GAACTGCTCCCGCTCGAC**
AMK26_RS11660-rev	**CGTACCCGGTGCAGTACG**

Red labeled are the overlapping regions with pOJ260, pSET152 and homologous arm.

Table S3. The quality of CB03234-G, CB03234-S and CB03234-GS26 genome sequence reads.

**Strains**	**Clean_Reads**	**Clean_Base**	**Clean Data Q20 (%)**	**Clean Data Q30 (%)**	**Mapping rate (%)**	**Average sequencing depth**
CB03234-G	11031949	3302803198	97.42	93.93	99.99	330
CB03234-S	7288750	2183844784	98.07	94.63	92.34	354
CB03234-GS26	11005419	3298389368	97.88	94.17	92.34	354

Fig.S1 Sanger sequencing results of mutations found in CB03234-G, CB03234-S and CB03234-GS26.

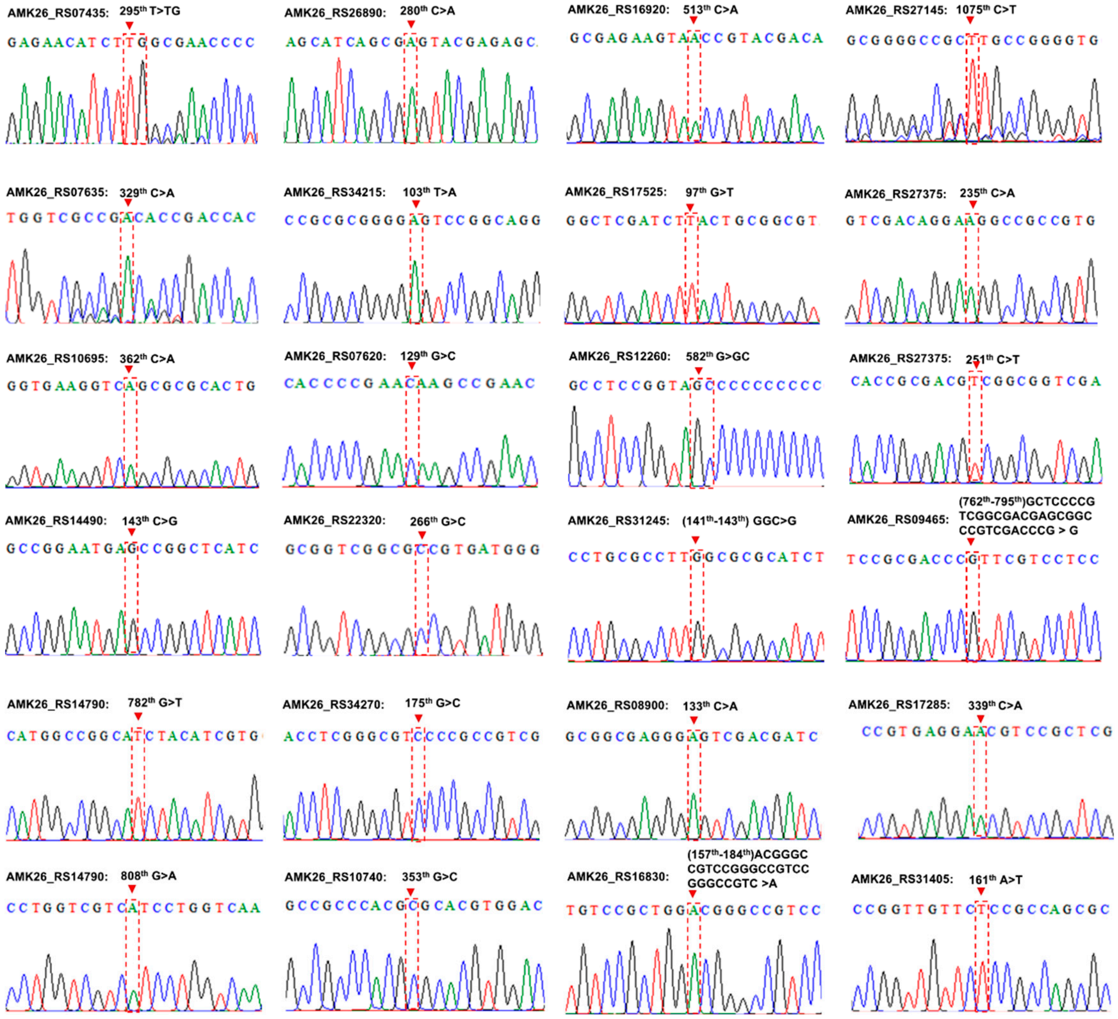

Fig.S2 CD analyses of four target proteins from CB03234-GS26 with the locations of their mutation sites.

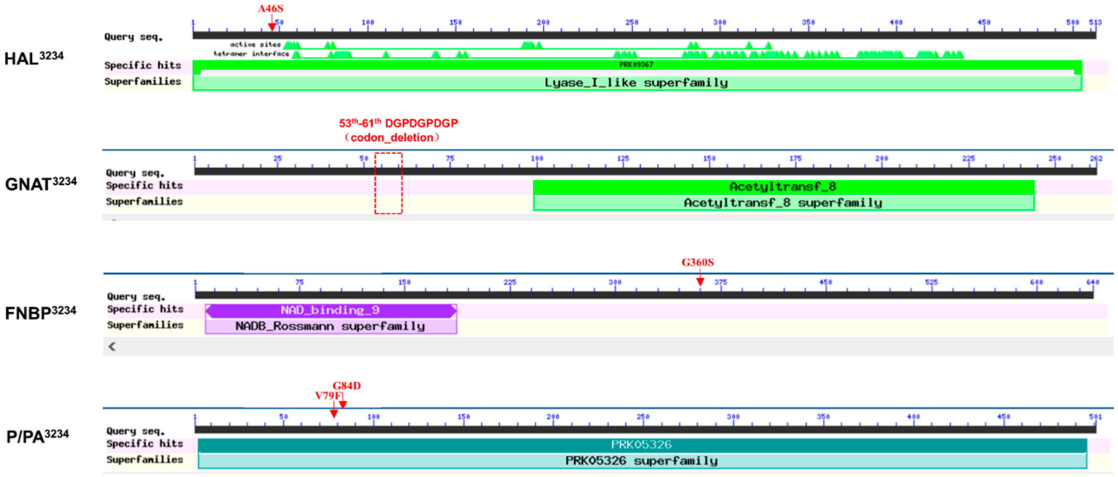

Fig.S3 Construction and PCR validation of gene knockout mutants derived from CB03234-S. **a** Knockout of *HAL*^{3234}. **b** Knockout of *GNAT*^{3234}. **c** Knockout of *FNBP*^{3234}. **d** Knockout of *P/PA*^{3234}.

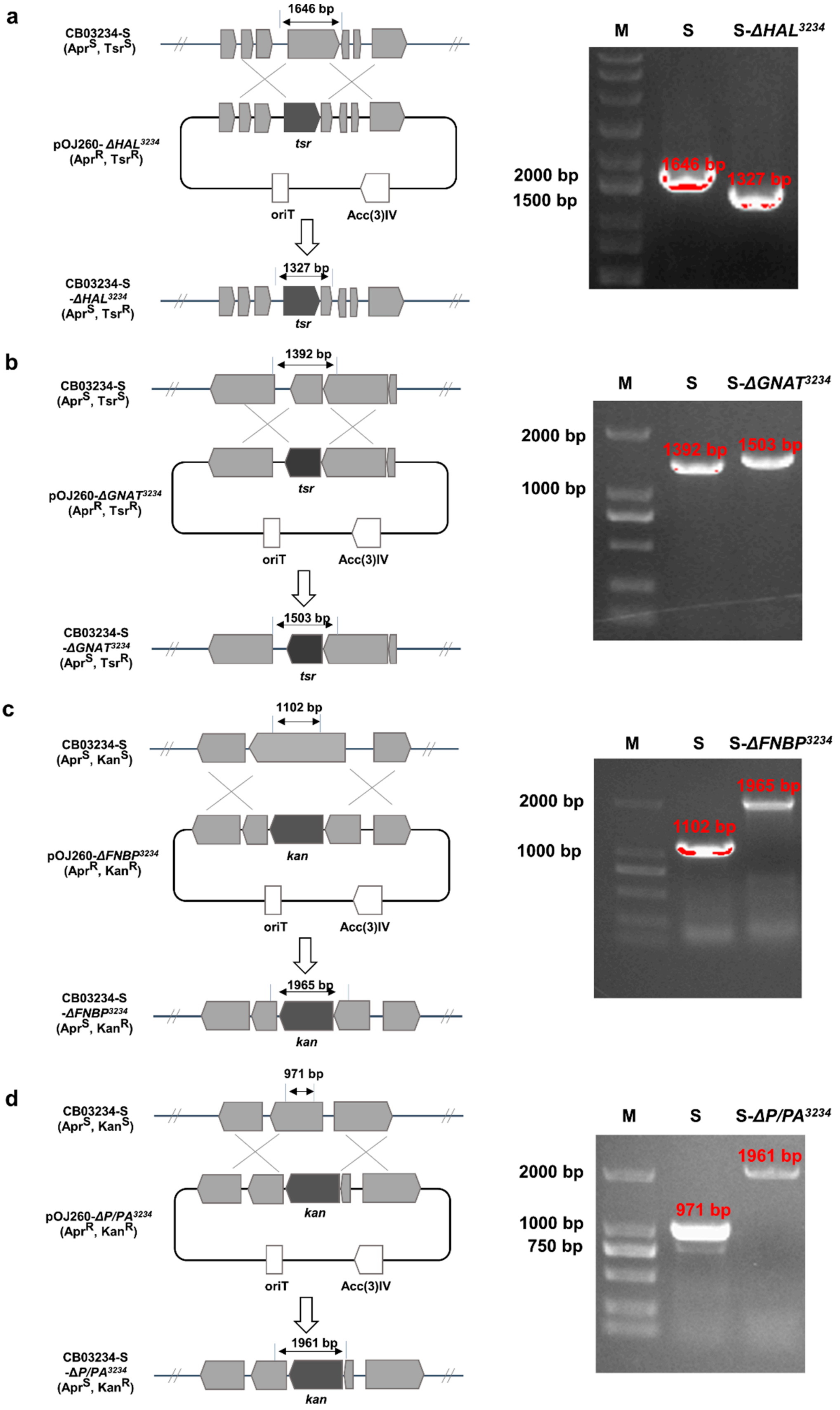

References

1. Simon, R.; Priefer, U.; Pühler, A. 1983 A broad host range mobilization system for in vivo genetic engineering: transposon mutagenesis in gram negative bacteria. *Nature* **1983**, *1*, 784–791. doi: 10.1038/nbt1183-784.
2. Yan, X.; Ge, H.; Huang, T.; Hindra; Yang, D.; Teng, Q.; Crnovcic, I.; Li, X., et al. Strain prioritization and genome mining for enediyne natural products. *mBio* **2016**, *7*, e02104–e02116. doi:10.1128/mBio.02104-16.
3. Zhuang, Z.; Jiang, C.; Zhang, F.; Huang, R.; Yi, L.; Huang, Y.; Yan, X.; Duan, Y., et al. *Streptomycin*-induced ribosome engineering complemented with fermentation optimization for enhanced production of 10-membered enediynes tiancimycin-A and tiancimycin-D. *Biotechnol. Bioeng.* **2019**, *116*, 1304–1314. doi: 10.1002/bit.26944.
4. Liu, H.; Jiang, C.; Lin, J.; Zhuang, Z.; Kong, W.; Liu, L.; Huang, Y.; Duan, Y., et al. Genome shuffling based on different types of ribosome engineering mutants for enhanced production of 10-membered enediyne tiancimycin-A. *Appl. Microbiol. Biotechnol.* **2020**, *104*, 4359–4369. doi: 10.1007/s00253-020-10583-2.
5. Bierman, M.; Logan, R.; O'Brien, K.; Seno, E. T.; Rao, R. N.;Schoner, B. E. Plasmid cloning vectors for the conjugal transfer of DNA from *Escherichia coli* to *Streptomyces* spp. *Gene* **1992**, *116*, 43–49. doi: 10.1016/0378-1119(92)90627-2.
